# Entropic Profiler – detection of conservation in genomes using information theory

**DOI:** 10.1186/1756-0500-2-72

**Published:** 2009-05-05

**Authors:** Francisco Fernandes, Ana T Freitas, Jonas S Almeida, Susana Vinga

**Affiliations:** 1 Instituto de Engenharia de Sistemas e Computadores: Investigação e Desenvolvimento (INESC-ID), R Alves Redol 9, 1000-029 Lisboa, Portugal; 2 Instituto Superior Técnico – Universidade Técnica de Lisboa (IST/UTL), Av Rovisco Pais, 1049-001 Lisboa, Portugal; 3 Dept Biostat Appl Math, Univ Texas MDAnderson Cancer Center – unit 447, 1515 Holcombe Blvd, Houston TX 77030-4009, USA; 4 Dept Bioestatística e Informática, Faculdade de Ciências Médicas – Universidade Nova de Lisboa (FCM/UNL), Campo Mártires da Pátria 130, 1169-056 Lisboa, Portugal

## Abstract

**Background:**

In the last decades, with the successive availability of whole genome sequences, many research efforts have been made to mathematically model DNA. Entropic Profiles (EP) were proposed recently as a new measure of continuous entropy of genome sequences. EP represent local information plots related to DNA randomness and are based on information theory and statistical concepts. They express the weighed relative abundance of motifs for each position in genomes. Their study is very relevant because under or over-representation segments are often associated with significant biological meaning.

**Findings:**

The Entropic Profiler application here presented is a new tool designed to detect and extract under and over-represented DNA segments in genomes by using EP. It allows its computation in a very efficient way by recurring to improved algorithms and data structures, which include modified suffix trees. Available through a web interface  and as downloadable source code, it allows to study positions and to search for motifs inside the whole sequence or within a specified range. DNA sequences can be entered from different sources, including FASTA files, pre-loaded examples or resuming a previously saved work. Besides the EP value plots, p-values and z-scores for each motif are also computed, along with the Chaos Game Representation of the sequence.

**Conclusion:**

EP are directly related with the statistical significance of motifs and can be considered as a new method to extract and classify significant regions in genomes and estimate local scales in DNA. The present implementation establishes an efficient and useful tool for whole genome analysis.

## Findings

### Algorithm

In a recent paper [1], the authors presented the concept of Entropic Profiles (EP), a new method to extract and classify relevant and statistically significant segments of DNA sequence. The study of these motifs is very relevant because under or over-representation segments are often associated with significant biological meaning.

The concept of Entropic Profiles was proposed previously but in a different context and distinct scope [2]. In that work, EP were calculated using the Shannon entropies of the histograms obtained from the Chaos Game Representation (CGR) [3] for different resolutions, i.e., using different L-tuples. This procedure allowed the discrimination between random and natural DNA sequences. Although the same name was used, this previous endeavor focused on a global perspective of sequence entropy [4] whereas the present work investigates a local entropy formulation. Another type of sequence profile also previously explored was based on Sequence Logos [5], giving the information content per position. However, this procedure required the alignment of a set of sequences and therefore is not an individual-based, alignment-free approach [6,7] like the present one.

Another type of sequence profile also explored was based on linguistic complexity [8] and low entropy DNA zones [9]. More recently, compression schemes were used to define Information Sequences (IS) [10] and the linear space complexity obtained allows exploring DNA. This algorithm uses a Lempel-Ziv-like compression algorithm to keep track of the repetitions, which can be very efficiently implemented by using suffix trees [11], also present in Entropic Profiler. However, this information content approach does not take into account neither explores the fact that a repetition can occur at different depths/lengths simultaneously since it uses a fixed low-order Markov model, geometric distribution and global set of probabilities for the beginning and ending of the repetitions. Entropic profiles, on the other hand, can automatically detect the best length to use in each position and also allow the study of a specific resolution while providing at the same time an overview of a range of lengths through a 3D plot. In this sense EP represent a combinatorial and exact approach, whereas IS are based in given compression schemes and Bayesian statistical models.

In this work EP plots express the relative abundance of corresponding motifs for each position and allow estimating local relevant scales. Its calculation is based on the continuous Rényi quadratic entropy [12], using the Parzen window estimation method [13] applied to the CGR of a sequence [3]. For each position, the EP function retrieves information about the L-tuple suffixes directly from the density kernel function, which allows the extraction of scale independent motifs.

To our knowledge, there are no current methods that calculate local information plots looking just at one individual sequence. The local scale given by the EP are estimated from the complete unique sequence and therefore can putatively have unbiased information about particular positions in genomes, without using any additional external information. The closest method is related to the calculation of the statistical significance of motifs, given as p-values or z-scores [14].

The main EP function [1] is defined by:

(1)

where *L *is the length resolution chosen, *ϕ *is a smoothing parameter, and *c*([*i*-*k*+1, *i*]) is the number of motifs (*x*_*i*-*k*+1 _... *x*_*i*_) in the whole sequence *x*_1 _... *x*_*N*_, i.e., the number of occurrences of the substring of length *k *that ends at position *i *in the sequence. This function can be interpreted as a linear combination of suffix counts up to a given memory length *L*, with increasing and decreasing weights (values of *ϕ*). It retrieves, for each position in the sequence, information about the abundance of the corresponding L-tuple suffix inside the entire sequence. To allow the comparison of different parameter combinations EP values must be further normalized by mean *m*_*L*, *ϕ *_and standard deviation *s*_*L*, *ϕ *_using all positions *i *= 1,..., *N*:

(2)

The computation (Eq. 1 and 2) is very time consuming because obtaining *m*_*L*, *ϕ *_and *s*_*L*, *ϕ *_involves calculating the EP values for all the sequence positions and all the calculations have to be executed several times and repeated for each value of *L *considered.

Along with this new method [1], the authors supplied a computational application developed in MATLAB to demonstrate their approach. Although the command-line based executables were also provided, the prototype version of this application provided a very poor performance, with excessive running times and memory usage. These restrictions severely hamper the usability of entropic profiles in practical situations, e.g., whole genome analysis. Furthermore, no additional information was provided regarding the comparison with other methods.

In the present implementation all the formulas were restructured and subject to several optimizations, which included the use of new data structures and extensive algebraic simplifications (see Supplementary Material). The result was a highly efficient web-based application, now described.

### Implementation

#### • Data structures

**Figure 1:**
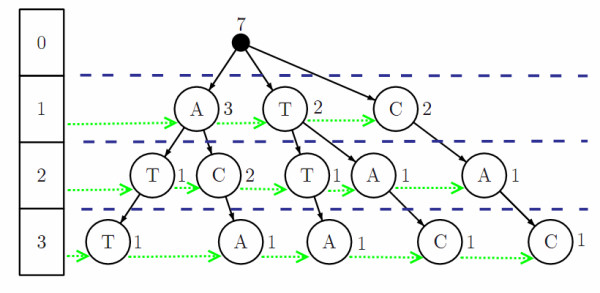
**Suffix tree and side links**. Example of a suffix tree and side links for word "ATTACAC" showing the suffix counts limited to depth 3. All substrings of length 3 are represented in the tree ("ATT", "TTA", "TAC", "ACA", "CAC"). Also shown are the side links connecting nodes of the same depth.

The major computational challenge in the core EP function corresponds to operations over suffixes with limited lengths. To improve these computations an efficient limited depth suffix tree structure [15] that stores the counts of each suffix was developed (Figure 1). The depth limit prevents the tree from growing without bounds, reducing time and memory consumption. The word counts are progressively updated during the building of the tree, allowing knowing the exact number of occurrences of a particular suffix inside the whole sequence just by a simple word matching operation over the suffix tree.

Furthermore all nodes, in the tree, at the same depth are connected through "side links" which are very useful to speed up some calculations.

This procedure is similar to creating a k-truncated suffix tree [16-18], which is demonstrated to efficiently searching for specific strings of length of up to *k *characters in large sequences. The present proposal has the difference that each node is labeled with a single symbol, so it resembles more to a trie than to a tree. Although this makes its construction a little slower and less compact, the speed improvement of this structure is noticed when we need the counts of all substrings of the same length, efficiently retrieved using the "depth-links".

#### • Algebraic and algorithm simplifications

The major improvement in all the simplifications on the formulas in Eq. 1 and Eq. 2 is that one can scan through all the motifs of a particular length inside the suffix tree instead of scanning through all the positions inside the whole sequence. The number of distinct motifs of length *L *is definitely much smaller than the size of the sequence. The suffix tree allows the indexation of all the L-tuple words and all their corresponding suffixes, for easy retrieval, and stores all their counts by scanning through the sequence only once. On the other hand, the "side links" allow to traverse the suffix tree horizontally and cover the counts of all words of the same length in a single sweep instead of searching the whole sequence over and over again.

Because DNA sequences can be extremely large, the search procedure for the positions of a particular motif has to be very efficient: the fast exact string matching algorithm "Shift-And" [19] was used for this purpose. Its great speed comes from the fact that all the operations required are implemented using basic arithmetic bitwise operations. All of these algorithms and data structures combined offer a fast, solid and robust backbone for the Entropic Profiler application.

#### • Statistical significance of motifs

In order to allow the comparison of the EP values with other statistical efforts, p-values and z-scores of the analyzed motifs are also reported. Over-represented motifs have a very low p-value and very high z-scores and EP values. These values are calculated using first-order Markov chain transition probability tables estimated directly from the whole sequence taking into account the overlap capacity or period of each motif [14].

#### • Interface

The Entropic Profiler tool is presented through an intuitive and easy to use web interface. The user can input a sequence by copy/paste the text or by uploading a FASTA file format. The sequence is saved so the user can later return to previous work. Pre-loaded examples are also available. The study can then be performed by selecting a particular position or a specific motif.

By default the results are displayed to the web browser. If the job takes more than 30 seconds the user is informed that an email will be sent with a link to the results, which will be kept by the system for five days. The Entropic Profiler tool provides different plots that allow the user to visually inspect information about all the motifs of distinct lengths within a given range, in a way that the most relevant ones are easily detected. EP values and detailed statistical data are also provided for a more precise reference. All the information can be downloaded as text files for subsequent study.

### Testing

The Entropic Profiler was tested in several DNA sequences, and the results for two genomes will be described below as two distinct examples of application. In particular, the user input options will be presented and the corresponding output will be described. After each example the user can click on the "back" button and recover the previous job for further analysis.

#### 1) Position study – Escherichia coli genome

When studying a position, the motif length can be chosen or the application can automatically determine the best one to use. A window length can also be specified to study a determined range of bp around the position.

**Figure 2:**
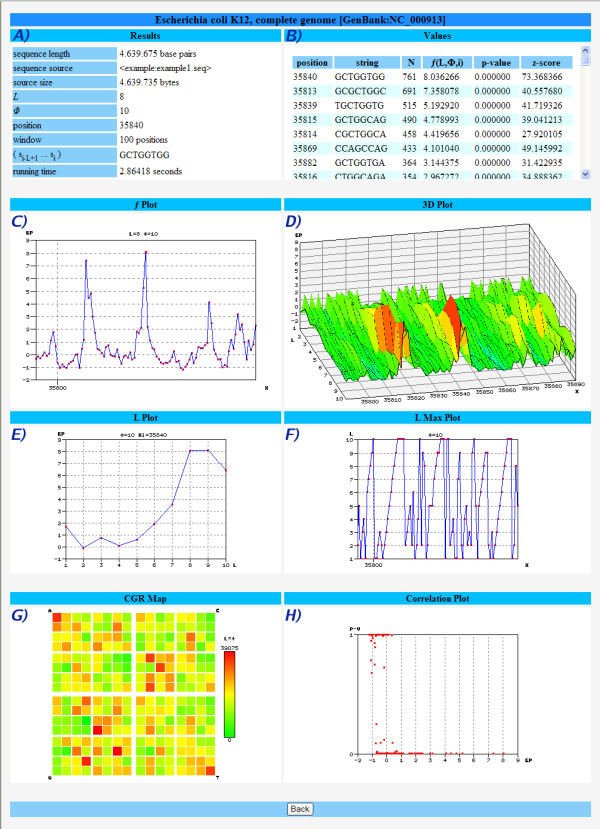
**Study sequence by position**. Example of the input and output screens of the Entropic Profiler application while studying around a position in the *Escherichia coli *K12 genome.

Figure 2 shows the output results for a pre-loaded example of the *Escherichia coli K12 *genome which uses as (default) input parameters the values *L *= 8, *ϕ *= 10, position 35840 and a window length of 100. Panel A) has the input values given from the user, for reference. In panel B) the listing of the all the motifs (string) with EP values *f*(*L*, *ϕ*, *i*) are given in decreasing order, along with their positions in the sequence and the corresponding p-values and z-scores. The user can download all the table information by clicking in the corresponding button, repeat the analysis for other position (click on the desired one) or perform a study by motif (see next example). Panel C) represents the EP plot along the range positions previously defined, where higher values correspond to the most significant suffixes that end in that position. Some information appears by pointing the mouse to each figure. Panel D) is the 3D plot of the same information but allowing for the variation of parameter *L*, the resolution. From this, one can analyze the relative increase of EP and visually identify the most promising *L *values that will lead to longer or more significant motifs. Panel E) plots the EP values for one particular position as a function of *L*, showing that there are values that maximize (or minimize) the EP, i.e., which correspond precisely to a local scale or the most distinctive suffix, when compared with the overall sequence. This allows to chose the "best" *L *for that particular position, which can be analyzed further. Panel F) calculates the value *L*, for each position in the defined range, which maximizes EP, which represents the local scale of each position. Panel G) is the CGR map of the complete sequence for resolution 4, which shows the relative density of strings. Finally, panel H) is a scatter plot that represents the relation between EP and p-values for the collection of motifs in the specified range.

A further look at this output shows that at position 35840 a highly rated motif of length 8 can be found (*5'-GCTGGTGG-3'*), which corresponds to a Chi site (crossover hotspot instigator), a key region that modulates the exonuclease activity of RecBCD (an enzyme that is necessary for chromosomal dsDNA repair and integration of exogenous dsDNA) [20-22].

It is noteworthy that this position can be obtained by analyzing the values *L *that maximize EP, without a previous knowledge of the length of the motif under study. In fact, each individual position corresponds to a local scale given by the resolution *L *that maximizes EP in that position, which equals exactly *L *= 8, the length of the biologically relevant motif.

#### 2) Motif Study – Haemophilus influenza genome

When studying the distribution of a specific motif, the application finds all the occurrences of the motif in the whole sequence or within a defined search range and plots the corresponding histogram in the submitted strand.

**Figure 3:**
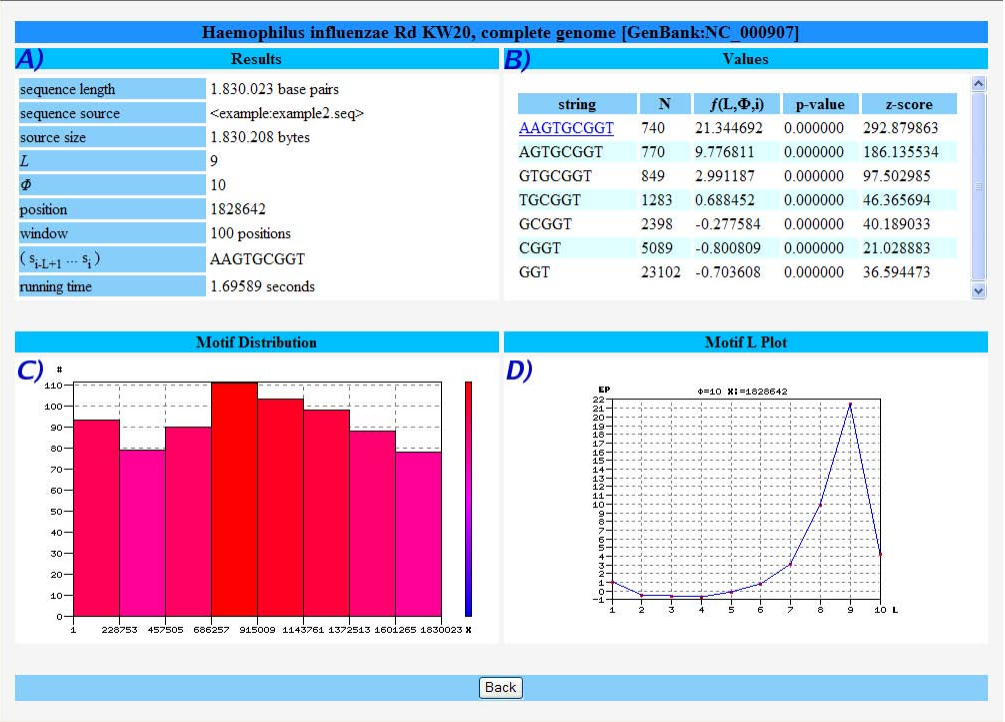
**Study sequence by motif**. Example of the input and output screens of the Entropic Profiler application while searching for motif "AAGTGCGGT" on *H. influenzae*.

Figure 3 shows the analysis for the well known motif "AAGTGCGGT" [23] in *Haemophilus influenza*, which represents a uptake signal sequence (USS+). USSs are involved in natural competence, which is a genetically controlled form of horizontal gene transfer in some bacterial species, related to their ability to take up DNA from the surrounding environment (reviewed in [24]).

Panel A) tables the input values given by the user. Panel B) represents the information for the input motif, along with all its sub-motifs down to length 3 (GGT in this case); the EP, p-values and z-score are calculated and the user can further click on each string to get the list of all positions where that string appears in the sequence (first symbol). All the information is available for download. These positions can be further studied by using the procedure above described in Example 1. The histogram of the occurrences of the motif is represented in panel C) and can be used to analyze the distribution of the positions of occurrences, shown to be an important feature of biologically significant motifs [25]. Finally, panel D) analyses one of the positions of occurrence in terms of the EP values vs. parameter *L*, which allows the determination of important resolutions for that particular string.

If the length of the motif is not known in advance, the user can search for particular maxima along the sequence – these positions can be further analyzed and the corresponding *L *retrieved, after which a more meticulous analysis can be conducted for those local scales.

#### • Performance

The average processing speed is around 2 seconds per million of basepairs (running on a machine with an Intel Xeon Quad-core CPU @ 1.60 GHz with 8 GB RAM). When loading a previous job or example these times are greatly decreased since the necessary data structures are already built and stored.

The current web application allows a maximum file size of 5 MB due to PHP and webserver constraints. For longer sequences a local version of Entropic Profiler should be used, by downloading the source code available at the webpage.

The examples provided above are for short bacterial genomes. This was in order to test Entropic Profiler in the analysis of known biologically relevant motifs. However, the analysis of large genomes can pose several problems, such as memory usage. In this context, compressed suffix arrays constructed with Burrows-Wheeler Transform were previously used to create indexes which allow efficient analysis [26,27]. Since Entropic Profiler uses suffixes trees which are limited in depth, a memory-wise efficient solution is also obtained, as described above.

In order to assess the performance of the tool for larger genomes, we also ran the algorithm for several other species several orders longer. The results, in terms of speed and memory, are in Table 1.

**Table 1:** Performance of Entropic Profiler for larger genomes.

**Name**	**Length** **(Mbases)**	**Time** **(s)**	**Memory** **(MB)**
*Caenorhabditis elegans*	97.6	75	171

*Homo sapiens *(CHR1)	239.2	164	290

*Takifugu rubripes*	382.7	263	411

*Gallus gallus*	940.1	611	1016

*Danio rerio*	1491.0	960	1567

For each species name, the length of each sequence tested (in 10^6 ^bases), the total running time (in seconds) and the memory usage (megabytes) are reported.

Note: The sequences can be assessed in the following links: Caenorhabditis elegans ; Homo sapiens (CHR1) ; Takifugu rubripes ; Gallus gallus ; Danio rerio .

As the results show, the additional space used beyond the size of the sequence is almost unnoticeable. This is explained by the fact that the suffix tree structure used has a limited depth, therefore it does not grow exponentially.

Although no biological analysis was performed in this situation, which would be out of the scope of this manuscript, these results demonstrate the feasibility of this type of analysis for large genomes and validate Entropic Profiler application in more challenging data.

### Future directions

Possible improvements to be implemented include the possibility to search for motifs in IUPAC code and the option to analyze both the forward and reverse strand of a sequence. The choice of programming language and web-based deployment was geared towards the future development of a web-based application with a simple Representational State Transfer (REST) I/O. As a consequence, the functionality described in this report will be also easily accessible as a URL POST which enables using the application as a web service.

## Conclusion

This work focused on the development of an efficient tool available through a web interface to compute the entropic profiles values for given DNA sequences. Since the main expression used to compute them is a function of suffix counts, the use of *suffix trees *to represent the sequences turned to be the major performance boost over the previous application.

Thanks to the developed side-links structure, calculations that involved searching the whole sequence over and over again to retrieve the counts of words of a particular length are now replaced by a single horizontal scan of the suffix tree at the depth corresponding to that specific word length. Some of the accomplished ideas, algorithms or structures developed for this particular work can also be reused and later readapted for building future efficient applications in similar sequence analysis projects. Besides the EP value plots, the p-value and z-score for each motif are also computed, along with the Chaos Game Representation of the sequence. The ability to load the sequence from different sources and to restore the last work also improves greatly the usability of the application. Its structured web-based presentation gave it a more appealing look and its intuitive interface made its use extremely straightforward. All of this makes the described application a fast, efficient and extremely useful tool for sequence and motif analysis that can be further expanded and combined with other algorithms.

## Availability and requirements

**Project name**: Entropic Profiler

**Project home page**: 

**Operating system(s)**: Platform independent

**Programming language**: C

**Other requirements**: None

**License**: None

**Any restrictions to use by non-academics**: None

## Competing interests

The authors declare that they have no competing interests.

## Authors' contributions

FF designed and implemented the algorithm optimizations and data structures and coded the application and the web interface. ATF and SV introduced the idea of applying suffix trees to EPs and provided insights and suggestions to improve the application. SV and JSA introduced the EP theory and guided the application development process. All authors contributed for the writing, read and approved the final manuscript.
